# Phase-Domain Peak-Based Correspondence Extraction for Robust Structured-Light Imaging

**DOI:** 10.3390/jimaging12050182

**Published:** 2026-04-23

**Authors:** Andrijana Ćurković, Milan Ćurković, Alen Grebo

**Affiliations:** 1 Faculty of Science, University of Split, 21000 Split, Croatia; 2 Faculty of Electrical Engineering, Mechanical Engineering and Naval Architecture, University of Split, 21000 Split, Croatia; milan.curkovic@fesb.hr (M.Ć.); alen.grebo.00@fesb.hr (A.G.)

**Keywords:** structured light, fringe projection, phase-shifting, phase images, peak detection, reflective objects, illumination variations

## Abstract

Standard fringe-based structured-light processing estimates wrapped phase from phase-shifted sinusoidal images and commonly relies on phase unwrapping to obtain a globally consistent phase representation. In practical measurements, this approach may become unstable on reflective objects and under low or non-uniform illumination, where the recorded fringe signal is distorted and the recovered phase becomes unreliable. To address these limitations, we propose a correspondence extraction method based on subpixel peak localization performed directly on phase-domain images. The wrapped phase is transformed into absolute value phase profiles, $\Phi =\left|\phi_{w}\right|$, whose local structure follows the projected fringe pattern and is less affected by object-dependent intensity variations. The proposed method reformulates correspondence extraction as a local signal-based estimation problem in the phase-domain, thereby reducing reliance on global phase-consistency constraints at the correspondence stage. A practical advantage observed in the evaluated examples is that the method remained usable in some regions where the phase became locally flat because of low modulation, saturation, or reflective surface effects. In such regions, conventional processing relies on sufficiently reliable phase gradients and subsequent unwrapping, whereas the proposed method uses local peak geometry in the transformed phase representation. In the implementation used here, Gray-code information is employed only for pixel-wise phase extension and reference indexing, not as a spatial phase-unwrapping mechanism. The method does not require machine learning models or training data and can be integrated as a correspondence analysis stage in practical structured-light systems.

## 1. Introduction

Structured-light imaging estimates object geometry from the deformation of projected patterns and establishes correspondences between projector and camera observations [1,2,3,4,5]. Fringe projection profilometry is widely used because phase-shifting techniques provide dense measurements and high accuracy under controlled conditions [6]. In the standard pipeline, several phase-shifted images are used to estimate wrapped phase, which is then unwrapped to obtain a globally consistent phase representation [6,7].

Despite its effectiveness, this pipeline remains sensitive to practical measurement conditions. Reflective surfaces, non-uniform illumination, saturation, and locally reduced fringe modulation degrade wrapped-phase quality and introduce ambiguities in phase unwrapping. Because unwrapping enforces global consistency, local phase errors may propagate over larger regions and cause loss of reliable correspondences. This remains one of the main difficulties in practical structured-light measurements.

Here, we propose a correspondence extraction method for structured-light imaging based on phase-domain representations. Rather than relying on global phase continuity as the primary correspondence mechanism, correspondences are determined by subpixel peak localization in the phase-domain. In the proposed framework, correspondence extraction is treated as a local signal-analysis problem in the phase-domain rather than as a global phase-reconstruction problem.

The central idea is that peak localization is driven by local extremum geometry of the transformed phase profile, which in the Rioux–Blais formulation is related to local second-order structure. In the evaluated examples, this proved useful in some regions where global phase variation became less reliable because of low modulation, saturation, or reflectance effects. The key distinction is that conventional pipelines rely on first-order phase variation, whereas the proposed method exploits second-order local structure for correspondence extraction.

The proposed approach does not remove noise, saturation, or dynamic-range limitations from the phase estimate itself. Instead, it changes how these disturbances affect correspondence extraction by emphasizing local peak geometry over global phase continuity. In the implementation used here, Gray-code patterns are applied on the calibration plane to establish reference correspondence locations and to assign pixel-wise fringe-order labels independently of spatial phase continuity. The main contribution of the paper, however, is the peak-extraction procedure itself. Gray-code information is used only for indexing and does not influence the local peak-detection mechanism.

The proposed framework should therefore be viewed as an alternative correspondence procedure based on phase-domain representations. It is not intended to replace established Gray-code-assisted or unwrapping-based methods in general use. Its purpose is to provide a simple and transparent local estimation procedure with favorable behavior in regions affected by reflection, low modulation, or non-uniform illumination.

It should be emphasized that the proposed method does not eliminate the need for phase indexing or phase extension in practical systems. When auxiliary coding (e.g., Gray-code patterns) is used to establish fringe order or reference correspondence, incorrect indexing may still affect the final result. The contribution of the method is therefore restricted to the local correspondence-extraction stage, not to replace phase indexing or phase recovery mechanisms.

### 1.1. Contributions

The main contributions of this paper are as follows:

- A structured-light correspondence analysis approach based on subpixel peak detection performed directly on phase-domain images.
- A wrapped-phase absolute value transformation that converts wrapped phase into peak-shaped profiles suitable for subpixel localization while reducing the influence of object-dependent intensity variations.
- A local correspondence formulation on phase-domain representations, where phase-domain images rather than raw intensity images serve as the working signal for the proposed detection stage.
- A comparison with conventional reference processing, together with discrepancy visualizations, qualitative consistency analysis, and a quantitative summary table.
- An extended evaluation including false-peak suppression criteria, processing-time comparison, and explicit discussion of method limitations.
- A clarification that auxiliary Gray-code information is used only for pixel-wise phase extension and reference indexing, not as part of the local phase-domain peak-detection principle.

### 1.2. Scope and Plan of the Paper

The rest of this paper is structured as follows. Section 2 outlines related work and the robustness limitations of conventional wrapped-phase processing. Section 3 describes the acquisition setup, the seven-step phase-shifting model, the phase-domain representation, and the peak-detection procedure. Section 4 reports the results, including qualitative examples, comparisons with reference methods, and runtime measurements. Section 5 discusses the interpretation of the results and the limitations of the method. Section 6 concludes the paper.

## 2. Related Work

Structured-light imaging and pattern codification strategies are reviewed in [1,2,3,4,5]. Gray-code-assisted phase-shifting approaches are also well established in structured-light imaging [8,9]. These methods combine coded patterns with phase measurements in order to improve phase-order estimation and indexing. Temporal unwrapping methods and robustness limits of conventional phase-processing pipelines are discussed in [6], while general phase-unwrapping theory is covered in [7]. These methods are effective under favorable conditions, but their reliability remains strongly dependent on the local quality of the wrapped phase.

Subpixel peak detection is widely used in optical profiling. The Rioux–Blais estimator provides a computationally inexpensive quadratic subpixel localization from three samples [10]. Robust structured-light approaches have also used local peak analysis for correspondence extraction [11], typically in the intensity domain. In contrast, the present paper applies peak detection on phase-domain absolute value phase profiles in order to reduce sensitivity to object texture and reflectance-driven intensity changes.

Another relevant line of work is based on directional-field analysis and local orientation estimation [12,13]. These methods process the local directional structure or spatial spectrum of fringed images and are of interest when fringe direction or frequency varies across the field. They differ from the present work, which does not estimate directional fields but instead transforms wrapped phase into a representation suited for local peak-based correspondence extraction.

Deep-learning-based structured-light systems can estimate phase or other measurement-related outputs from fewer patterns [4,14,15,16]. The method proposed here is a direct classical solution that does not require machine learning models or training data. In that sense, it can also serve as a transparent reference stage for evaluating and analyzing learning-based structured-light methods.

### Robustness Limitations of the Wrapped-Phase Pipeline

The reliability of the standard phase-shifting and phase-unwrapping pipeline is strongly dependent on the local quality of the wrapped phase. Comprehensive reviews [3,4,5,6] highlight that specular reflection, locally reduced fringe modulation, saturation, and depth discontinuities degrade wrapped-phase accuracy and can trigger error propagation during unwrapping. Experimental studies on saturated fringes [17] and high dynamic-range measurement scenarios [18,19] further demonstrate that reflectance variation and intensity clipping introduce phase distortions that are difficult to fully compensate for within conventional wrapped-phase workflows. Likewise, applications in biomedical and industrial environments report degraded measurement reliability when fringe contrast is locally reduced or when illumination conditions vary significantly [20,21,22,23].

These observations motivate the present work. The proposed method does not attempt to improve the wrapped-phase estimate itself. Instead, it changes the correspondence-extraction stage so that the final result depends on local peak geometry in a transformed phase signal rather than on successful global phase recovery.

## 3. Materials and Methods

### 3.1. Acquisition Setup and Data

**Figure 1:**
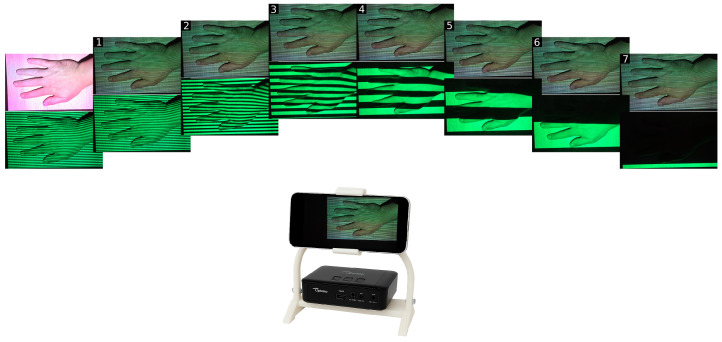
Example acquisition environment. The complete set may include a reference view, seven same-frequency phase-shifted sinusoidal patterns used for phase computation, and additional Gray-code patterns used only for auxiliary coarse decoding.

A structured-light scanner typically consists of a calibrated camera–projector pair. In the implementation used here, the measurement system includes a single camera and a projected-pattern source rather than a dual-camera stereo configuration. A calibration plane is used as the reference surface for establishing projector–camera correspondence. Gray-code patterns are employed on the calibration plane to identify corresponding projected fringe locations, while peak positions are computed on both the calibration plane and the measured object. In this way, the calibration-plane peaks provide the reference correspondence structure against which the object-surface peaks are analyzed. The acquisition set includes a reference image, *N* sinusoidal phase-shifted fringe images (here N=7), and optionally Gray-code patterns for coarse projector-coordinate decoding and indexing. Figure 1 shows an example acquisition set.

The upper part of Figure 1 summarizes the captured sinusoidal and coded patterns used in the acquisition sequence, while the lower image shows a representative reference view of the measured object. This figure clarifies that the proposed method starts from a standard multi-image fringe acquisition and does not require a special machine learning-oriented input representation. The seven sinusoidal patterns used in the proposed method have the same carrier frequency and differ only in phase shift. The additional coded patterns shown in Figure 1 are not part of the seven-step phase computation and are included only to illustrate the complete acquisition environment of the scanner. This clarification is important because the overview figure combines sinusoidal and coded patterns, whereas the mathematical model in Equations (1)–(5) is restricted to the seven same-frequency sinusoidal images.

In the notation of Equation (1), $I_{0}$ denotes the direct-current (DC) component of the observed intensity.

The present paper focuses on the seven sinusoidal images and on phase-domain representations used for peak-based correspondence analysis. The peak-detection stage itself does not rely on spatial or temporal phase unwrapping. In the illustrated measurement setup, auxiliary coded information is used only for phase extension and reference indexing, not as part of the local peak-localization principle.

### 3.2. Algorithmic Workflow

The complete procedure can be summarized as a sequence of signal-domain transformations and local estimations. Starting from the seven captured fringe images, the method first computes the wrapped phase, then forms a phase-domain representation, transforms it into absolute value phase images, extracts one-dimensional peak profiles, and localizes peaks with subpixel precision.

**Figure 2:**
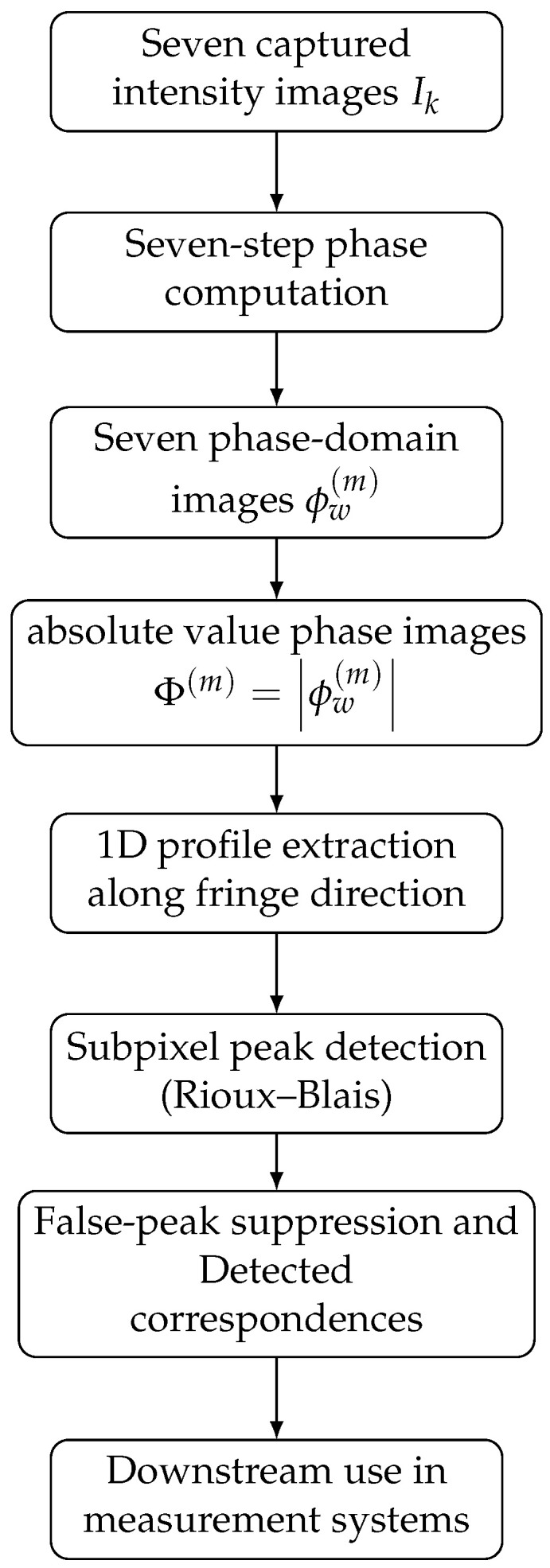
Schematic overview of the proposed algorithm. The method operates on phase-domain representations and their absolute value phase counterparts before peak localization.

Figure 2 illustrates this workflow in compact form. The figure emphasizes that the method does not insert a machine learning stage and does not depend on any learned latent representation. Instead, the entire analysis is carried out through an explicit sequence of phase computation, phase-domain representation formation, and subpixel localization. The detected peak positions can then be used as robust structured-light correspondences in downstream measurement systems, but those downstream stages are outside the scope of this paper.

### 3.3. Seven-Step Phase-Shifting and Wrapped Phase

For an *N*-step phase-shifting algorithm, the captured intensity at pixel (x,y) in the *k*-th image is modeled as

(1) $$\begin{array}{cc} I_{k}\left(x,y\right)\; & =I_{0}\left(x,y\right)+I_{m}\left(x,y\right)\cos\;\left(\phi \left(x,y\right)+\delta_{k}\right),\\ \; & \;\;k=1,\ldots ,N, \end{array}$$

where $I_{0}$ is the DC intensity, $I_{m}$ is modulation, ϕ(x,y) is the object-dependent phase, and $\delta_{k}$ are phase steps. For a uniform seven-step scheme,

(2) $$\delta_{k}=\frac{2\pi }{7}\left(k-1\right),\;k=1,\ldots ,7.$$

Wrapped phase is computed using harmonic sums [3,6]:

(3) $$\begin{array}{cc} C(x,y)\; & =\sum_{k=1}^{7}I_{k}\left(x,y\right)\cos\;\left(\frac{2\pi }{7}\left(k-1\right)\right), \end{array}$$

(4) $$\begin{array}{cc} S(x,y)\; & =\sum_{k=1}^{7}I_{k}\left(x,y\right)\sin\;\left(\frac{2\pi }{7}\left(k-1\right)\right), \end{array}$$

(5) $$\phi_{w}\left(x,y\right)=\mathrm{atan}2\;\left(-S(x,y),\;C(x,y)\right),\;\phi_{w}\in \left(-\pi ,\pi \right].$$

In the implementation used here, auxiliary Gray-code patterns are applied on the calibration plane to establish reference correspondence between projected fringes and camera observations and to determine an integer fringe-order term at each pixel. This coded information is combined with the wrapped phase to form an absolute phase representation through pixel-wise phase extension. Because the fringe-order term is obtained independently from the coded patterns rather than from spatial phase continuity, this procedure differs fundamentally from classical spatial phase unwrapping and is referred to here as Gray-code-assisted phase extension.

Equation (5) gives the standard wrapped phase associated with the original ordering of the seven captured images. However, because the seven-step phase-shifting sequence is cyclic, the same acquisition can also be represented through seven cyclic phase references. Each cyclic representation is obtained by circularly shifting the phase-step indices prior to phase evaluation. Accordingly, instead of working with only one wrapped-phase image, we form a stack of seven phase-domain images

(6) $$\phi_{w}^{\left(m\right)}\left(x,y\right),\;m=1,\ldots ,7,$$

where each $\phi_{w}^{\left(m\right)}$ corresponds to one cyclic permutation of the seven-step phase sequence. For the proposed method, these cyclic phase-domain images are not a replacement for phase computation. They serve as the working representation for the subsequent local correspondence extraction stage. These cyclic representations preserve the same acquisition data while shifting the local phase reference, which changes the alignment of peak-shaped structures used in the subsequent detection stage. Because the phase-shifting sequence is cyclic, each cyclic permutation produces a phase-domain representation with slightly different local peak alignment. This property improves peak detectability and robustness in regions where local profile quality varies across the image. In practice, this redundancy increases the probability that at least one cyclic phase-domain representation exhibits a sufficiently well-defined local peak structure for stable localization. In the following, several intermediate phase representations are shown. Their purpose is to illustrate the signal used in the proposed procedure, not to introduce an additional phase-recovery step.

**Figure 3:**
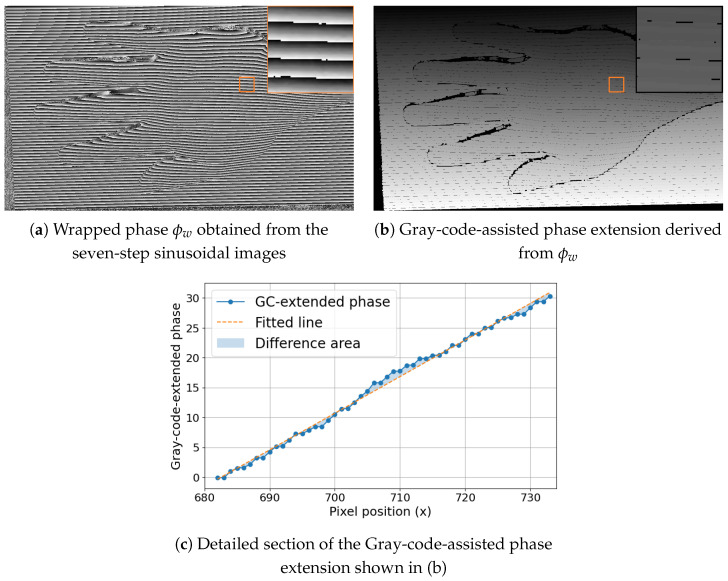
Wrapped phase (**a**), Gray-code-assisted phase extension (**b**), and a magnified local section of the extended phase representation obtained from the seven-step sinusoidal acquisition (**c**).

Figure 3 illustrates several intermediate representations obtained after the seven-step phase computation. Figure 3a shows the wrapped phase obtained directly from the captured seven-image set, Figure 3b shows the corresponding Gray-code-assisted phase extension, and Figure 3c shows a detailed local section of the extended phase image. Together, these subfigures indicate that the phase signal is not perfectly smooth in practical measurements. Instead, it contains local disturbances caused by reflectance variation, noise, specular highlights, and reduced contrast, which motivates the use of a signal-processing strategy that does not depend on stable global unwrapping.

In Figure 3b, dark pixels may be visible in some regions. These pixels do not indicate missing phase values in the theoretical sense. Instead, they correspond to low-confidence regions identified during implementation, where additional consistency checks based on auxiliary coded patterns detect locally inconsistent phase labels. Such pixels are therefore excluded from further processing and should be interpreted as rejected measurements rather than as a limitation of the phase computation itself.

### 3.4. Conventional Phase-Shifting Reference Processing

For comparison, we consider a conventional processing chain based on wrapped phase computation, modulation analysis, and phase unwrapping. This reference follows the standard fringe projection profilometry workflow up to the stage of obtaining a globally consistent phase description [3,6,7]. Any subsequent system-specific use of that representation is treated here only as a downstream black-box stage and is not part of the paper.

#### 3.4.1. Wrapped Phase and Modulation Quality

Using the seven-step phase-shifting model in (1)–(5), we compute the wrapped phase $\phi_{w}\left(x,y\right)\in \left(-\pi ,\pi \right]$. A commonly used data-quality indicator is the fringe modulation amplitude (up to a constant factor),

(7) $$A\left(x,y\right)=\sqrt{C^{2}\left(x,y\right)+S^{2}\left(x,y\right)},$$

where *C* and *S* are the harmonic sums defined above. Low values of *A* typically indicate shadowed regions, saturation, or reduced fringe contrast, which correlates with increased phase noise and unwrapping failures.

Optionally, a validity mask can be formed as

(8) $$\Omega =\{\left(x,y\right)\;|\;A\left(x,y\right)\geq \tau_{A}\},$$

with a threshold $\tau_{A}$ chosen empirically. In the comparison in Section 4.2, we avoid aggressive masking in order to expose failure regions in the conventional processing chain.

#### 3.4.2. Phase Unwrapping and Unwrapped Reference Signal

The goal of phase-order recovery is to determine an integer fringe order k(x,y)∈Z such that the absolute phase can be written as

(9) $$\phi_{u}\left(x,y\right)=\phi_{w}\left(x,y\right)+2\pi \;k\left(x,y\right),$$

with $\phi_{u}$ consistent with the underlying fringe geometry, up to a global constant.

The estimation of k(x,y) depends on the chosen strategy. In spatial phase unwrapping, k(x,y) is recovered from local phase continuity by applying an operator of the form

(10) $$k\left(x,y\right)=U\left(\phi_{w}\left(x,y\right)\right),$$

where U(·) denotes a spatial unwrapping procedure [6,7]. Under low modulation, noise, or specular distortions, such methods may introduce incorrect 2π jumps that propagate over larger regions.

In contrast, temporal or coded approaches (e.g., Gray-code-based methods) estimate k(x,y) from additional pattern sequences rather than from spatial phase continuity. In these cases, k(x,y) is not a function of $\phi_{w}\left(x,y\right)$ alone, but is obtained independently from auxiliary measurements.

The proposed method does not rely on spatial phase unwrapping for local correspondence extraction. While the experimental setup may employ Gray-code-assisted phase extension to obtain indexing information or an absolute phase representation for reference purposes, this information is not part of the local peak-detection principle itself. Instead, the method operates on phase-domain representations and performs local correspondence extraction by subpixel peak localization.

#### 3.4.3. Relation to the Proposed Peak-Based Method

The conventional pipeline represents the scene through global phase consistency and a continuous unwrapped phase map (9). In contrast, the proposed method extracts local subpixel correspondences from absolute value phase representations derived from the wrapped phase. Therefore, the reference approach depends strongly on successful unwrapping or fringe-order estimation, whereas the proposed approach evaluates local correspondence primarily through local peak structure and peak detectability after phase formation.

### 3.5. Absolute Value Phase Profiles for Peak Detection

Rather than relying on global phase continuity for correspondence extraction, we transform the wrapped phase into an absolute value phase representation. For each phase-domain image $\phi_{w}^{\left(m\right)}\left(x,y\right)$, we define the corresponding absolute value phase image

(11) $$\Phi^{\left(m\right)}\left(x,y\right)=\left|\phi_{w}^{\left(m\right)}\left(x,y\right)\right|,\;m=1,\ldots ,7.$$

For simplicity, when discussing local profile behavior, the notation

(12) $$\Phi \left(x,y\right)=|\phi_{w}\left(x,y\right)|$$

will refer to one representative member of this absolute value phase stack.

The role of the transformation $\Phi =\left|\phi_{w}\right|$ is not to create new phase information but to reorganize the wrapped phase into a representation with symmetric local peak structures. From a signal-processing perspective, the transformation converts zero-crossing structures of the wrapped phase into symmetric extrema. In the principal wrapped interval (−π,π], sign changes around zero-crossings cause local profile reversals that are inconvenient for direct peak localization. Taking the absolute value removes this sign ambiguity and produces a peak-centered representation that is well suited for local quadratic estimation. The transformation should therefore be understood as a signal reparameterization for local correspondence extraction rather than as a new phase-recovery step.

If the phase were represented in the interval [0,2π), the absolute value operation would have little practical effect because the sign ambiguity would already be absent. The proposed transformation is therefore specifically meaningful for the standard wrapped representation in (−π,π].

Peak detection can in principle be performed either on the original intensity images or on phase-domain profiles. In the present work, it is performed on the absolute value phase representation $\Phi =\left|\phi_{w}\right|$, not on the original intensity images. This distinction is important because peaks in the raw intensity data are strongly affected by surface texture, reflectance, shadows, and local illumination changes, whereas phase-domain profiles follow the projected fringe structure more directly.

In the standard seven-step phase-shifting acquisition, the processing pipeline starts from seven captured intensity images

(13) $$I_{1}\left(x,y\right),I_{2}\left(x,y\right),\ldots ,I_{7}\left(x,y\right),$$

which contain both projected fringe information and object-dependent photometric effects such as reflectance variation and illumination changes. After applying the phase-shifting operator in Equation (5), the original intensity stack is no longer used as the primary signal for correspondence extraction. Instead, the working representation is transferred into the phase-domain.

In the implementation considered here, the original sequence is represented by the corresponding stack of seven phase-domain images in Equation (6), which serve as the signal used for further analysis. These phase-domain images encode primarily the projected fringe geometry rather than the raw intensity structure of the object surface. As a result, they provide a more stable representation for peak extraction in difficult measurement conditions.

Applying the magnitude transformation in Equation (11) converts the wrapped phase into peak-shaped phase profiles that are well suited for local subpixel peak localization. Consequently, the processing chain operates on phase structures that are less affected by object appearance and illumination conditions than the original intensity images.

In practice, Φ reduces sensitivity to object texture and reflectance-driven intensity variations compared with using raw intensity profiles, because it is derived from the phase-shifting operator rather than from local intensity maxima. Consequently, peak localization is driven primarily by the projected fringe structure. Even when reflective regions reduce modulation or apparent phase slope, the absolute value phase profile often preserves sufficient local structure for peak estimation, as discussed later in Section 3.9.

**Figure 4:**
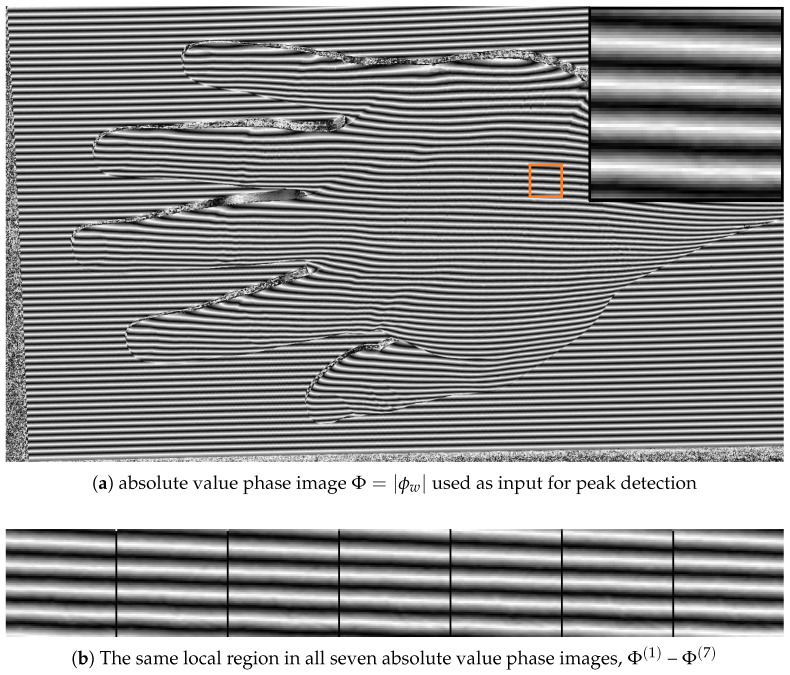
Example phase-domain absolute value image for peak detection (**a**), and the same local region across all absolute value phase images (**b**).

Figure 4 shows how the wrapped phase is transformed into the absolute value phase-domain. Figure 4a presents an example image $\Phi =\left|\phi_{w}\right|$ that serves as the direct input for peak detection, while Figure 4b shows the same local region across all seven absolute value phase images. These images illustrate that the transformation does not merely visualize the wrapped phase differently, but reorganizes it into a form that produces more regular local peak structures.

**Figure 5:**
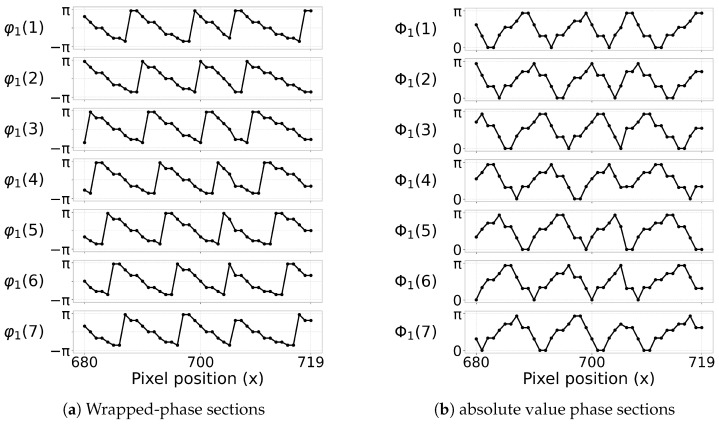
Sections from a wrapped-phase profile (**a**) and from the corresponding absolute value phase profile (**b**).

Figure 5 gives a more local view of the same idea. The Figure 5a shows representative sections extracted from the wrapped-phase image stack, while the Figure 5b shows the corresponding sections after the magnitude transformation. The main visual difference is that the wrapped-phase sections exhibit sign changes and profile reversals, whereas the absolute value phase sections produce a clearer peak-shaped structure. This is precisely the property exploited by the subpixel peak detector in the next subsection.

### 3.6. Subpixel Peak Estimation

For three samples $f_{i-1},\;f_{i},\;f_{i+1}$ around a discrete maximum, the Rioux–Blais estimator [10] gives the subpixel peak position

(14) $$x_{p}=i+\frac{f_{i-1}-f_{i+1}}{2\left(f_{i-1}-2f_{i}+f_{i+1}\right)}.$$

Here $f_{i}$ are samples extracted from one-dimensional profiles of Φ along the fringe direction. Equation (14) shows that peak localization depends on local profile shape in a very small neighborhood, which makes the method computationally simple and well suited to phase-domain profiles that already exhibit peak-like behavior after the transformation in (12).

In this work, the Rioux–Blais estimator is used as the local correspondence mechanism itself. The estimate is obtained directly from the extremum of the transformed phase profile and does not follow a separate similarity-based matching step.

### 3.7. False-Peak Suppression

In non-ideal conditions, one-dimensional profiles extracted from Φ may contain spurious local maxima. To reduce false detections, the proposed method uses three practical constraints. First, a candidate peak must exceed a local amplitude threshold relative to the neighboring profile values. Second, the discrete curvature term *D* in Equation (16) must remain above a minimum threshold, which rejects very flat or noisy extrema. Third, the candidate is checked for consistency across neighboring cyclic phase-domain images $\Phi^{\left(m\right)}$, which helps suppress isolated accidental maxima.

Let $f_{i}$ denote the sampled one-dimensional profile. A peak candidate at index *i* is accepted only if

(15) $$f_{i}\geq \tau_{f},\;\left|D\right|\geq \tau_{D}$$

where $\tau_{f}$ and $\tau_{D}$ are empirical thresholds, and if a corresponding local maximum is observed in at least one neighboring cyclic representation. In the implementation used here, the threshold values are reported as $\tau_{f}=0.8\pi$ and $\tau_{D}=0.1$. These values were chosen empirically and remained unchanged across all evaluated examples.

### 3.8. Output Representation and Downstream Use

Each detected peak $x_{p}$ defines a local correspondence in the phase-domain representation associated with a stable projected fringe event. In the context of this paper, the output of the proposed method is the set of such subpixel correspondences extracted from the phase-domain image stack. These correspondences can be passed to downstream system-specific stages, but the present work is limited to the signal-processing side, namely formation of the phase-domain representation and robust subpixel localization on that representation.

To preserve generality and avoid dependence on any particular proprietary implementation, subsequent geometric or application-specific processing is treated here only through illustrative downstream visualizations. The emphasis of the paper is therefore on the stability, detectability, and interpretability of the detected peaks in the phase-domain representation.

### 3.9. Peak Detectability in Locally Flattened Phase Regions

Reflective areas and locally disturbed fringe contrast can produce regions where the wrapped phase $\phi_{w}\left(x,y\right)$ varies slowly along the nominal fringe direction. In conventional pipelines, reduced or unstable phase gradients can lead to unreliable unwrapping and loss of valid signal structure.

**Figure 6:**
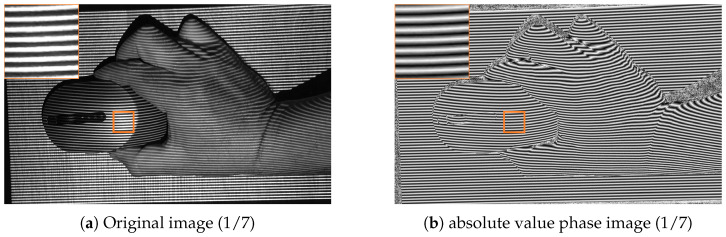
Comparison of an original captured image (**a**) and the corresponding absolute value phase image (**b**) used for phase-domain peak extraction.

Figure 6 compares a captured original image with its corresponding absolute value phase image in a region affected by reflection. The original image contains both projected fringe information and object-dependent photometric structure. In contrast, the absolute value phase image suppresses much of the object-dependent variation and reveals a more regular profile structure suitable for peak extraction. This comparison helps explain why the proposed method operates on phase-domain representations rather than directly on intensity data.

In the proposed method, peak localization is performed on the absolute value phase representation $\Phi =\left|\phi_{w}\right|$ and depends on local second-order structure rather than on global phase slope. For a one-dimensional profile $f_{i}$ sampled from Φ around a discrete peak, the Rioux–Blais estimator is governed by the discrete curvature term

(16) $$D=f_{i-1}-2f_{i}+f_{i+1}.$$

When |D| is sufficiently above the noise level within the local window, subpixel peak estimation remains well conditioned even if the large-scale phase slope is small. Empirically, reflective regions often attenuate modulation and reduce apparent gradients, but they do not necessarily eliminate the local peak curvature in Φ, which enables stable peak localization.

This difference summarizes the practical motivation of the method. Conventional unwrapping-based processing relies strongly on reliable first-order phase variation, whereas the proposed method uses local second-order structure of the transformed phase signal for correspondence extraction.

This interpretation can also be formulated in terms of signal requirements. Conventional phase-unwrapping pipelines rely on sufficiently strong and consistent first-order phase variation, which may be degraded under low modulation, reflection, or saturation. In contrast, the proposed method requires that the local phase-derived profile retains sufficient second-order structure for peak localization. In the evaluated examples, this condition was sometimes satisfied even when the phase gradient became unreliable, allowing correspondence extraction in regions where conventional methods degraded.

**Figure 7:**
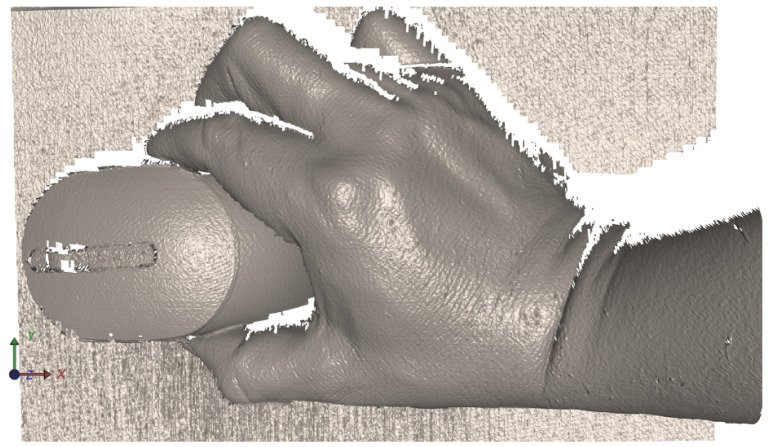
Illustrative downstream visualization derived from phase-domain correspondences extracted by the proposed approach for the example in Figure 6, including regions with locally flattened phase due to reflective surface behavior.

The following example (Figure 7) demonstrates a case with locally flattened regions caused by specular reflection and strong highlights, where usable downstream signal structure can still be extracted by the proposed method. The figure should be interpreted as an illustrative downstream visualization rather than as a central methodological component of the paper. The important observation is that the detected phase-domain correspondences remain sufficiently stable in zones where a gradient-based unwrapping pipeline would typically degrade.

This interpretation should be understood as an empirical observation based on the evaluated examples rather than as a general analytical result. While local curvature may remain detectable in some degraded regions where phase gradients become unreliable, this does not imply that second-order structure is universally more robust than first-order variation. A formal analysis of this behavior remains outside the scope of the present work.

## 4. Results

### 4.1. Qualitative Examples

**Figure 8:**
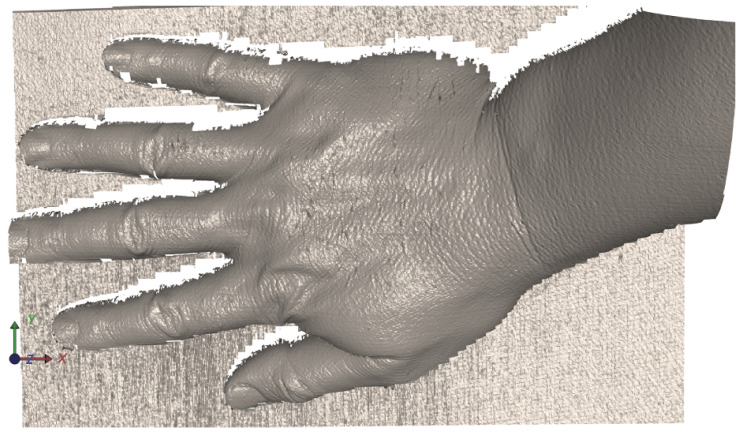
Illustrative downstream visualization obtained by applying the proposed peak-based procedure to the absolute value phase image $\Phi =\left|\phi_{w}\right|$.

The qualitative results support the main premise of the proposed method, namely that the absolute value phase representation provides local peak structures that remain sufficiently stable even in visually problematic regions. The examples shown in Figure 7 and Figure 8 indicate that the extracted correspondences preserve usable signal structure not only in regular regions but also in regions affected by reflections and local phase flattening. These examples therefore support the view that peak extraction on phase-domain images can act as a robust alternative to standard unwrapping-dependent signal processing.

The algorithmic sequence shown in Figure 2 also helps interpret these results. The output quality does not depend on recovering one globally correct unwrapped phase field, but rather on repeated detection of reliable local peak events in the phase-domain image stack. In that sense, the qualitative visualizations presented here are consistent with the signal-processing logic of the proposed approach.

### 4.2. Comparison with Conventional Phase-Shifting and Unwrapping Reference Processing

To compare the proposed peak analysis on phase-domain images with a conventional phase-shifting pipeline (Section 3.4), we analyze two outputs: (i) the set of correspondences obtained from the proposed peak detector operating on phase-domain images, and (ii) the output produced by the conventional phase-shifting and phase-unwrapping reference processing chain.

Because the two outputs do not necessarily have one-to-one correspondence, we evaluate their discrepancy using a nearest-neighbor distance field in the downstream comparison space. Here, X and Y denote 3D points in the compared downstream point-cloud representations obtained from the proposed and reference processing chains, respectively. For each point X from the proposed output, we define

(17) $$d\left(X\right)=\min_{Y\in P_{\mathrm{ref}}}{∥X-Y∥}_{2},$$

where $P_{\mathrm{ref}}$ denotes the set produced by the reference processing chain. The reported distance map visualizes d(X) over the resulting downstream representation. Summary statistics can be computed as

(18) $$d_{50}={\mathrm{median}}_{X\in P_{\mathrm{prop}}}d\left(X\right),\;d_{90}={\mathrm{percentile}}_{90}d\left(X\right),$$

where $P_{\mathrm{prop}}$ denotes the set obtained by the proposed method. Thus, $d_{50}$ and $d_{90}$ represent geometric discrepancy measures computed on reconstructed 3D point sets, rather than local signal-based similarity measures.

**Figure 9:**
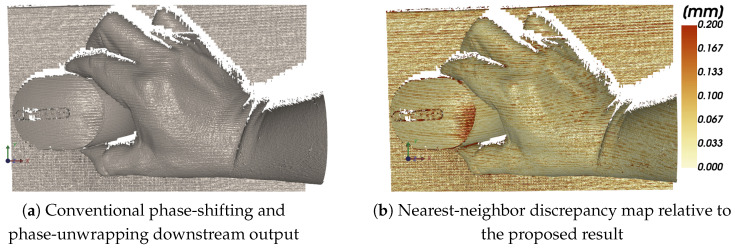
Comparisonbetween conventional reference processing (**a**) and the proposed peak-based approach on phase-domain images. The right image (**b**) visualizes the nearest-neighbor discrepancy field defined in (17).

Figure 9 presents this comparison. Figure 9a shows the output produced by conventional phase-shifting and phase-unwrapping downstream processing, while Figure 9b shows the nearest-neighbor discrepancy map relative to the proposed result. The reference output reveals the typical weaknesses of the conventional pipeline in difficult regions, namely local degradation and increased inconsistency. The discrepancy map makes these differences easier to interpret spatially by showing where the two outputs diverge the most.

### 4.3. Quantitative Summary

To complement the visual comparison, Table 1 reports the qualitative correspondence reliability together with nearest-neighbor discrepancy statistics for the compared methods.

**Figure 10:**
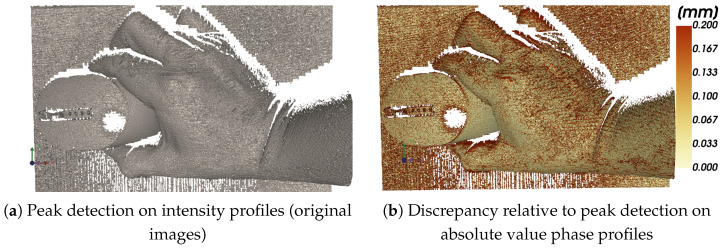
Comparison (**b**) of peak-based outputs obtained using intensity-domain peaks (**a**) and peaks on phase-domain images (absolute value phase profiles).

The term “Valid correspondences” qualitatively indicates how reliably each method produces usable and stable peak locations across the scene. As indicated by the discrepancy maps in Figure 9 and Figure 10, the conventional phase-shifting and phase-unwrapping pipeline shows localized disagreement with the proposed method, particularly in reflective and boundary regions. Quantitatively, this comparison yields a median discrepancy of $d_{50}\;=\;0.0625$ and a 90th percentile of $d_{90}=0.1629$.

In contrast, peak detection performed directly on the original intensity images exhibits substantially larger and more spatially widespread deviations ($d_{50}=0.1258$, $d_{90}=0.3729$), confirming that intensity-domain peaks are more sensitive to texture, illumination, and reflectance variations.

The proposed peak detection on phase-domain images serves as the internal reference in the present discrepancy evaluation. For that reason, $d_{50}$ and $d_{90}$ are not reported for this method in Table 1. If evaluated against itself, these values would be zero by construction and should not be interpreted as absolute accuracy or superiority.

The evaluation focuses on nearest-neighbor discrepancy measures because ground-truth correspondences are not available for the real-world data used here. The reported values therefore quantify consistency between competing outputs rather than absolute reconstruction accuracy.

### 4.4. Additional Quantitative Measures and Processing-Time Comparison

In addition to the discrepancy-based comparison, the evaluation also considered processing time because practical structured-light systems require not only robust correspondence extraction but also predictable runtime. The implementation was tested on a Samsung Galaxy S22+ smartphone (8-core CPU, 4 GB available RAM). For the proposed workflow, the total time required to generate the 3D surface and display the result on the screen is 1.94 s.

To make the comparison with conventional processing explicit, Table 2 summarizes the total execution times measured under the same hardware conditions. The proposed method includes phase computation, formation of cyclic phase-domain images, absolute value phase transformation, profile extraction, false-peak suppression, subpixel peak localization, correspondence matching, and visualization. The conventional reference method includes phase computation, phase-order recovery or unwrapping, downstream reconstruction, and visualization.

The two implementations are of the same order of magnitude in total runtime, but their computational structure is different. In the conventional pipeline, the phase-order recovery stage introduces additional costs and its behavior depends on the complexity and local consistency of the phase field. In the proposed workflow, the correspondence-extraction stage is based only on local operations. As a result, the runtime is more predictable and does not require additional global error-correction steps associated with spatial unwrapping.

Although the noise characteristics at the phase-estimation level are governed by the captured fringe data in both methods, the proposed strategy changes the error-propagation mechanism. In the conventional pipeline, local phase-order mistakes may spread to larger regions through global consistency constraints. In the proposed method, the final estimate is derived from local peak geometry, so the influence of local disturbances remains spatially confined.

### 4.5. Peak Detection on Intensity vs. Phase-Domain Profiles

The comparison in Figure 10 motivates peak extraction on phase-domain images. Peaks detected directly on intensity profiles are affected by object texture, spatially varying albedo, shadows, and specular highlights, which distort peak shape and bias subpixel localization. In contrast, phase-domain profiles are produced by the phase-shifting operator and therefore encode the projected fringe structure more directly. As a result, peak detection on $\Phi =\left|\phi_{w}\right|$ yields more stable and repeatable correspondences, especially in reflective regions where intensity-based peak localization may fail or become inconsistent.

Figure 10a shows an illustrative downstream result obtained when peaks are detected on original image data, while Figure 10b shows the discrepancy relative to the phase-domain result. This pair of images is useful because it separates the two sources of information present in structured-light data, namely raw captured brightness on one side and phase-domain fringe structure on the other. The visual comparison confirms that using phase-domain profiles reduces the effect of object appearance on the final detected correspondence set.

While the comparison in Figure 10 provides a global visual indication of the difference between intensity-domain and phase-domain peak extraction, a more focused local evaluation is needed in order to assess signal behavior under specific measurement conditions. For this reason, an additional analysis on selected regions of interest (ROIs) was performed using local signal-similarity measures.

### 4.6. Region-of-Interest Comparison with a Local Matching Baseline

To provide a more focused evaluation of the proposed local correspondence mechanism, additional comparisons were performed on selected regions of interest (ROIs). The selected ROIs were chosen as representative examples of different measurement conditions, including (i) regular regions with high fringe modulation and (ii) challenging regions affected by reflection or reduced contrast.

This ROI-based analysis should be distinguished from the earlier evaluation based on $d_{50}$ and $d_{90}$ in Section 4.2. Those measures quantify geometric discrepancy between reconstructed 3D outputs, whereas the present ROI analysis evaluates local signal similarity directly on corresponding one-dimensional peak neighborhoods.

In addition to the proposed peak detection on phase-domain images, a local matching baseline was implemented. This baseline corresponds to peak detection performed directly on intensity-domain profiles extracted from the original images. While simple, this approach represents a commonly used local correspondence strategy and provides a meaningful reference for evaluating the behavior of the proposed phase-domain representation.

Local signal similarity between corresponding peak neighborhoods was evaluated using the sum of squared differences (SSD)and normalized cross-correlation (NCC), which are widely used in image matching and template comparison [24,25]. SSD measures absolute differences between signals, while NCC evaluates normalized structural similarity and is less sensitive to amplitude variations.

For each ROI type (regular and reflective region), local comparisons were performed between the measured object and the reference calibration plane.

For each detected peak, a one-dimensional profile was extracted from the object and from the corresponding peak location on the reference plane. This procedure was carried out separately for phase-domain profiles (derived from $\Phi =\left|\phi_{w}\right|$) and for intensity-domain profiles (from the original captured images).

Each profile consisted of seven samples centered at the detected peak location (three samples on each side of the central peak). Local signal similarity between object and reference profiles was evaluated using the sum of squared differences (SSD) and normalized cross-correlation (NCC).

For each representation (phase-domain and intensity-domain) and each ROI (regular and reflective), the resulting SSD and NCC values were aggregated over all peak correspondences. The values reported in Table 3 correspond to the mean and median of these distributions.

**Figure 11:**
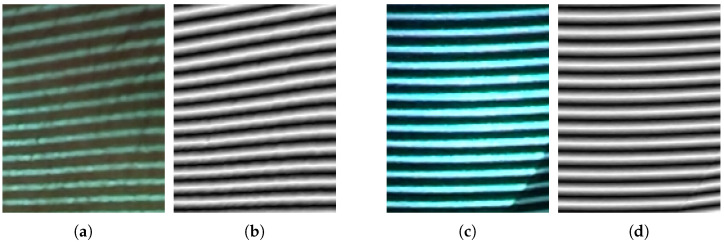
(**a**–**d**): intensity-domain and phase-domain views of a regular region, followed by intensity-domain and phase-domain views of a reflective region.

The quantitative results for representative ROIs are summarized in Table 3, while Figure 11 shows the corresponding regular and reflective ROIs in both intensity-domain and phase-domain representations.

The results indicate that phase-domain comparisons remain comparatively stable across the evaluated measurement conditions. Both SSD and NCC values exhibit minimal variation between regular and reflective regions, indicating that the phase representation preserves local signal structure even in the presence of reflectance effects.

In contrast, intensity-domain comparisons show clear degradation in reflective regions. SSD values increase significantly, while NCC values decrease, indicating loss of structural similarity caused by reflectance distortions, saturation, and illumination variations.

This ROI-based evaluation isolates the behavior of the proposed correspondence functional under both regular and reflective measurement conditions. The reported SSD and NCC values should be interpreted as relative indicators of local signal agreement between corresponding peak neighborhoods. The phase-domain representation is used here as a stable comparison signal, but it is not interpreted as ground truth.

This formulation allows a consistent comparison of local correspondence behavior without requiring external ground-truth data.

The ROI-based comparison indicates that the behavior of the two local correspondence strategies depends on the measurement conditions. In regular regions, both signal representations exhibit relatively stable local agreement with the reference. In reflective regions, however, phase-domain profiles remain comparatively stable, while intensity-domain profiles degrade significantly.

These observations support the interpretation that the absolute value phase representation provides a more robust local signal structure for peak localization under degraded measurement conditions. However, the observed improvement should be regarded as empirical and dependent on the specific signal characteristics within each ROI.

The ROI-based comparison is intended to isolate the effect of the local correspondence functional under controlled local conditions. It is not meant as an exhaustive benchmark against all local correspondence methods reported in the literature, and broader comparative evaluation remains future work.

## 5. Discussion

### 5.1. Why Peak Detection on Phase-Domain Images Improves Robustness

Standard fringe projection pipelines compute wrapped phase and recover a globally consistent signal through spatial or temporal unwrapping [6,7]. Their robustness relies on accurate wrapped phase and sufficiently consistent local phase gradients. Under specular reflection, saturation, and locally reduced modulation, wrapped-phase quality deteriorates and unwrapping may propagate local errors into large regions [3,6,17].

The main difference between the proposed method and conventional processing is in the correspondence stage. Standard phase-shifting pipelines recover a globally consistent phase representation and then use that representation in subsequent processing. The proposed method performs local correspondence extraction on transformed phase-domain profiles. As a result, its behavior at the correspondence stage is governed primarily by local peak detectability rather than by the stability of a global unwrapping process.

The method does not replace phase indexing or phase unwrapping where such stages are required, but introduces a different local correspondence functional applied to phase-domain representations. This reformulation shifts the focus from recovering a globally consistent phase field to identifying stable local geometric features in the phase signal. In conventional unwrapping-based pipelines, local phase-order errors may spread across extended regions. In the proposed method, disturbances remain localized because the final estimate is derived from local peak geometry rather than from global phase continuity. Peak localization depends on local second-order structure, that is curvature, rather than on global phase gradient. As discussed in Section 3.9, peak estimation remains stable as long as the discrete curvature term in (16) remains above the noise level.

This property becomes particularly important in regions where the phase becomes locally flat because of low modulation, saturation, or reflectance effects. In such cases, the phase gradient may be weak or unreliable, which directly limits the performance of gradient-based unwrapping methods. In contrast, the present method does not depend on strong phase variation and instead exploits local peak curvature in the absolute value phase representation. Even when the phase gradient is small, the local profile may still exhibit enough curvature for stable subpixel localization, which makes it possible to extract usable correspondences in otherwise problematic regions.

It should be noted that the method does not remove the underlying sources of measurement degradation, such as reflectance variations, noise, or dynamic-range limitations. Instead, it changes how these effects influence the final result. By avoiding dependence on global spatial phase continuity during correspondence extraction, the method reduces the risk that local disturbances influence larger image regions and tends to confine their impact to local peak estimates.

The sequence of figures in the Section 3 and Section 4 supports this interpretation. Figure 3 and Figure 4 show how the raw wrapped phase is converted into a more peak-oriented representation. Figure 2 formalizes the signal transition from the original image stack to the phase-domain stack and then to the absolute value phase stack used for peak extraction. Figure 5 and Figure 6 illustrate that the transformed phase contains more usable local peak geometry than the original image data. Figure 7, Figure 9 and Figure 10 then show how these local advantages are reflected in downstream visualizations.

Finally, because the method does not require training data or learned models, it provides a transparent reference pipeline that can be used to analyze and benchmark learning-based structured-light methods under the same acquisition conditions [4,14,15,16].

### 5.2. On Reference Processing and Comparisons

A direct comparison against conventional phase-shifting and phase-unwrapping processing is valuable when the acquisition follows standard sinusoidal projection assumptions. In this work, the reference chain is included in order to expose typical failure behavior under reflective and low-modulation conditions and to provide an interpretable discrepancy map, as shown in Section 4.2. The primary focus nevertheless remains the suitability of phase-domain profiles for robust peak extraction in practical measurement conditions.

The comparison with intensity-domain peak detection is equally important. It shows that the advantage of the proposed method does not arise only from using peak detection instead of unwrapping, but also from using phase-domain representations rather than the raw intensity domain as the signal on which peaks are estimated. This distinction is essential for interpreting the actual contribution of the work.

The proposed method is related to several established structured-light approaches, but differs from them in the correspondence-extraction stage. Gray-code-assisted phase-shifting methods [8,9] combine coded patterns with phase measurements in order to improve phase-order estimation and indexing. Classical phase-unwrapping methods [6,7] recover a continuous phase field from spatial or temporal consistency. Intensity-domain peak or feature-based methods [11] determine correspondences directly from image data and therefore remain sensitive to texture, reflectance variation, and illumination change. In contrast, the present method operates on phase-domain representations and determines correspondences by local peak localization in a transformed phase signal.

This discussion is intended to position the method with respect to related approaches, rather than to claim universal superiority or to provide a direct benchmark-style comparison with all alternatives. The goal of the comparison is to isolate the effect of the phase-domain representation rather than to benchmark against all local correspondence strategies. A fair direct benchmark would require shared datasets, matched hardware, and standardized evaluation protocols, which are outside the scope of the present study.

A direct experimental comparison with deep-learning-based structured-light methods would be relevant, but a fair benchmark would require controlled datasets, matched projector–camera hardware, and matched evaluation conditions. For that reason, the present work positions the proposed method as a classical, training-free reference strategy rather than claiming direct superiority over learning-based approaches. Future work will include broader benchmark-style comparisons against learning-based methods on shared evaluation settings and, where possible, on publicly available data.

### 5.3. Limitations

The proposed method is governed by local peak detectability and therefore depends on sufficient peak curvature in the absolute value phase profiles. If the local signal becomes extremely flat, heavily corrupted by noise, or strongly saturated, the Rioux–Blais estimator may become unstable.

An additional limitation is related to the narrow-projected fringe structures used in the acquisition. Because the projected sinusoidal tracks are relatively thin, their apparent contrast decreases when the surface moves away from the focal distance. In such regions the phase profiles become flatter and peak curvature is reduced, which can make peak localization less reliable. As a result, when a single scan is performed with fixed focus, the proposed method may tolerate a smaller depth variation in the object than conventional phase-shifting pipelines with wider fringe patterns.

This limitation is directly related to the correspondence-based nature of the method. The use of narrower phase tracks improves local peak separability, but it also reduces the admissible depth range along the optical axis. In practical terms, this means that objects with large variation along the *z*-axis may require multiple focus settings, modified fringe widths, or a reduced measurement volume.

A further limitation is related to surface roughness. Strong local roughness may perturb fringe modulation and reduce the regularity of the local peak profile, which can affect localization stability. The present paper does not model this effect explicitly, and its influence should therefore be regarded as an open practical limitation rather than as a quantified result.

A further limitation is the current scale of experimental validation. The present study is based mainly on controlled in-house data and illustrative downstream visualizations. Broader cross-dataset validation and expanded benchmark-style comparisons remain outside the scope of the present paper.

This work focuses on the signal-processing stage. Downstream geometric or application-specific processing is outside the scope of this paper.

The additional ROI-based comparison provides a more direct evaluation of the proposed method against a local correspondence baseline. This comparison complements the previous analysis based on global processing pipelines and clarifies that the contribution of the method lies in the local matching functional applied to phase-domain representations. A broader comparison against a wider range of local correspondence methods remains an important direction for future work.

## 6. Conclusions

The paper presents a peak-based correspondence extraction approach for structured-light imaging based on phase-domain images and transformed phase profiles. By transforming wrapped phase into absolute value phase peak profiles and using them as the primary working representation for the local correspondence extraction stage, the method reduces the influence of object-dependent intensity variations and supports robust correspondence extraction for reflective objects and under low or non-uniform illumination.

The presented qualitative and quantitative comparisons indicate that the proposed peak detection on phase-domain images provides more stable correspondence extraction than peak detection performed directly on the original intensity images. The comparison with conventional phase-shifting reference processing also shows that the proposed method remains usable in regions where the standard wrapped-phase pipeline becomes less reliable. Since the approach does not require machine learning models or training data, it may serve both as a practical signal-processing stage and as an interpretable benchmark for future structured-light research.

The main contribution of the study is the formulation of correspondence extraction as a local estimation procedure on phase-domain representations, based on transformed phase profiles and subpixel peak localization. The method is not presented as a new coding or phase-recovery scheme, but as a different local operating mode with favorable behavior under reflective surfaces, low modulation, and non-uniform illumination.

The study also clarifies the role of the absolute value transformation, the local nature of the correspondence strategy, the handling of false peaks, and the practical limitations related to narrow fringe tracks, depth variation, and surface roughness. Auxiliary Gray-code information, when used, is limited to pixel-wise phase extension and reference indexing. The method does not replace phase indexing or phase extension, but modifies the local correspondence functional applied after phase computation by operating on phase-domain representations. The timing analysis further indicates that the method provides predictable runtime because the correspondence-extraction stage relies on local operations after phase computation.

## Figures and Tables

**Table 1:** Qualitative and quantitative summary of the compared methods.

Method	Valid Correspondences	Median $d_{50}$	P90 $d_{90}$
Standard phase-shifting	Moderate	0.0625	0.1629
Intensity peak detection	Moderate, but unstable	0.1258	0.3729
Phase-domain peak detection	High	– (reference)	– (reference)

**Table 2:** Run time summary.

Method	Total Time (s)	Main Processing Stages
Conventional phase processing + unwrapping reference	2.56	phase computation, phase-order recovery/unwrapping, reconstruction, visualization
Proposed phase-domain peak method	1.94	phase computation, profile extraction, local peak detection, matching, visualization

**Table 3:** Comparison of local signal similarity using sum of squared differences (SSD) and normalized cross-correlation (NCC) for phase-domain and intensity-domain profiles in regular and reflective regions.

		SSD		NCC	
Representation	Region	Mean	Median	Mean	Median
Phase-domain	Reflective region	0.3788	0.2373	0.9129	0.9485
Phase-domain	Regular region	0.3675	0.2406	0.9137	0.9480
Intensity-domain	Reflective region	6.4991	6.2695	0.7105	0.7764
Intensity-domain	Regular region	2.9990	2.0791	0.7448	0.8228

## Data Availability

The original contributions presented in this study are included in the article. Further inquiries can be directed to the corresponding author.

## Abbreviations

The following abbreviations are used in this manuscript:

FPP	Fringe Projection Profilometry
PMP	Phase Measuring Profilometry
